# Medical Opinion and Movement

**Published:** 1907-08-03

**Authors:** 


### Medical Opinion and Movement.

Dr. E. Toff, of Munich, has made some useful suggestions in regard to the care of the perineum during labour. He points out that a considerable number of cases of ruptured perineum occur in spite of the usual methods of protecting the perineum, by supporting it with the hand and allowing the fœtal head to pass slowly through the vulvar opening. He considers that the determining cause of rupture is the disproportion between the parts passing through and also the inelasticity of the external genitals. The best protection for the perineum is to ensure, therefore, that the fœtal head passes through the vulva with its most favourable diameter, that is, the sub-occipito frontal, instead of the occipito frontal, or mento-occipital. In order to ensure this, extension of the head must be delayed as long as possible by pressing on the occiput. With the hand on the vulva and the fingers directed towards the symphysis, attempts should be made at each pain to bring down more of the occiput, and so increase flexion. Not until the whole occiput has passed below the symphysis should extension be allowed to take place. Toff has carried out this principle in a large number of cases during the last four years, and claims not to have had a single rupture.

The temerity with which new cancer "cures" have been heralded forth by certain of the daily papers has not without reason been somewhat severely criticised from time to time in the medical press. To such criticism our lay contemporaries have responded by representing the profession, together with its journals as almost hostile to the discovery and use of a new and effectual remedy for this dire disease. In view of the arduous struggles which members of the profession are carrying on in all parts of the globe to wrestle from Nature the secret which she has hitherto so closely held from us, such allegations are, of course, ridiculous. But to the "man in the street" those in authority, or in possession of technical knowledge, can always be successfully belittled. With a few plausible arguments it is not difficult to represent the profession as a most foolish and obstinate body of men. In spite, however, of all the booming which the trypsin treatment of cancer has received, its more extended trial since its first appearance has not confirmed the hopes that were entertained of its efficacy. Nevertheless there are signs and portents that we may be nearing the wished-for goal, and it seems not improbable that a weapon may be forged to fight the scourge sucessfully in the form of some ferment which, when inoculated, will have a specific power in arresting the growth of the cancer cell, and even destroying it.

Dr. Gordon Mackay has recently published a most interesting case of mammary scirrhus. After removal of the tumour recurrence took place, the disease progressed rapidly, involving the other breast, the axillæ, and both pleuræ, with extensive pleuritic effusions on both sides of the chest. For some weeks the patient was in a state of semi-collapse with laboured respiration and swallowing almost impossible, when suddenly the effusion was absorbed, and, together with this absorption, the whole condition rapidly cleared up, and the cancerous masses disappeared, and the patient regained weight and strength. Dr. Mackay connects this extraordinary recovery with the absorption of the pleuritic effusion, and suggests that the patient became inoculated with some virus in the effusion, which acted specifically upon the cells of the cancerous masses. It sounds plausible. If we can but discover the nature of this virus and the conditions of its formation, we may have here the key to the solution of the problem. We understand that Professor Bier is experimenting on the lines of serum therapy with pig's blood, while Professor E. von Leyden and Dr. P. Bergell, of Berlin, are carrying out investigations on the action of liver ferments on malignant growths. They found that the injection of a liver ferment into malignant growths was followed by rapid disintegration of the tissue cells, and that it was more selective for cancer tissue than radio-active substances or pancreatin. At present their reasearches are only in the first stage, but we trust that if any satisfactory results follow they will not be brought before the public, until they have been thoroughly tested and confirmed.

Volume I. of the appendix to the second interim report of the Royal Commission on Tuberculosis contains a full account of the extensive research work of Dr. Griffiths. This work has an important practical bearing upon the question of human infection with tuberculosis through the food supply. A large variety of animals, including adult bovines and calves, pigs, dogs, cats, anthropoid apes, monkeys, and others were treated with bovine tuberculous material of different sources and kinds, either by inoculation or feeding, and the results observed, chiefly by post-mortem examinations. One of the most important experiments consisted in the inoculation of a cow in full milk with tubercle culture, and the subsequent inoculation of guinea-pigs with the milk, with the result that they all developed tuberculosis, although the udders showed no signs of the disease, demonstrating what has already been contended by other observers, that the milk from a tuberculous cow may be infected, even though the udders are apparently healthy. The infection of animals by feeding with tuberculous material is of especial interest. Dr. Griffiths's experiments showed that both anthropoids and monkeys are very susceptible, and that the disease usually runs a progressive course. From the feeding experiments on these and other animals it appears that tubercle bacilli ingested may, within a very short time, find their way into the lungs, and whenever general tuberculosis results, the lungs are usually affected, often to a greater extent than any other organ of the body. These facts are obviously of the greatest importance in relation to the possible source of infection for human beings from the food, and should awaken the authorities to more energetic measures for the control and supervision of both the milk and the meat supply.

